# Co-regulation of Clustered and Neo-functionalized Genes in Plant-Specialized Metabolism

**DOI:** 10.3390/plants9050622

**Published:** 2020-05-13

**Authors:** Takayuki Tohge, Alisdair R. Fernie

**Affiliations:** 1 Graduate School of Biological Science, Nara Institute of Science and Technology (NAIST), Ikoma 630-0192, Japan; 2 Max Planck Institute of Molecular Plant Physiology, 14476 Potsdam-Golm, Germany

**Keywords:** plant specialized metabolism, gene cluster, tandem gene duplication, co-regulation network, neo-functionalization, metabolic evolution

## Abstract

Current findings of neighboring genes involved in plant specialized metabolism provide the genomic signatures of metabolic evolution. Two such genomic features, namely, (i) metabolic gene cluster and (ii) neo-functionalization of tandem gene duplications, represent key factors corresponding to the creation of metabolic diversity of plant specialized metabolism. So far, several terpenoid and alkaloid biosynthetic genes have been characterized with gene clusters in some plants. On the other hand, some modification genes involved in flavonoid and glucosinolate biosynthesis were found to arise via gene neo-functionalization. Although the occurrence of both types of metabolic evolution are different, the neighboring genes are generally regulated by the same or related regulation factors. Therefore, the translation-based approaches associated with genomics, and transcriptomics are able to be employed for functional genomics focusing on plant secondary metabolism. Here, we present a survey of the current understanding of neighboring genes involved in plant secondary metabolism. Additionally, a genomic overview of neighboring genes of four model plants and transcriptional co-expression network neighboring genes to detect metabolic gene clusters in Arabidopsis is provided. Finally, the insights functional genomics have provided concerning the evolution and mechanistic regulation of both the formation and operation of metabolic neighboring clusters is discussed.

## 1. Introduction

Plants produce a huge variety of specialized metabolites (secondary metabolites), which have been characterized as the defense metabolism of plants and the arsenals they develop come about via adaptation to and natural selection caused by ecological niches and environmental factors [1,2,3]. Generally, the chemical diversity of plant specialized metabolites has been greatly expanded, either by horizontal gene transfer from other species, or by functional diversification following tandem gene duplication during metabolic evolution [4,5,6]. Such gene duplication is often found as a key innovator of metabolic evolution in expanding the metabolic diversity of plant metabolism, including species-specific specialized metabolism. Species-specific neighboring genes, therefore, have been the focus of considerable research [7]. 

Metabolic gene clusters constructed by the genomic neighboring of biosynthetic genes were originally discovered in the operons of bacterial genomes [8], with operon-like gene clusters later also being found, for genes associated with primary and secondary metabolism, in plant genomes [6]. Metabolic gene clusters for terpenoid biosynthesis have been observed in several land plant species. So far, terpenoid biosynthesis in rice [9,10,11], tomato [12,13], oat [14], *Arabidopsis thaliana* [4,15,16], Brassica plant species [17] and *Lotus japonica* [18] are reported to contain such metabolic gene clusters [7,19]. Additionally, biosynthetic genes of isoquinoline alkaloids in opium poppy, bezoxazinones in maiz as well as cyanogenic glucosides in lotus, cassava and sorghum were found within metabolic gene clusters [19]. However, the core genes involved in land-plant conserved secondary metabolism, such as flavonoid and hydroxycinnamate pathways, were not yet found as a gene cluster in any plant species. Given that metabolic gene clusters have only been found in secondary metabolism which arose relatively recently after plant speciation, such gene clusters tend to be found in species-specific secondary metabolism. 

Neo-functionalization, by contrast, which has been occurred following tandem gene duplication and results in differential substrate specificity of the independent gene copies, is also identified as a key factor of metabolic evolution [7]. Following secondary metabolite analysis in *A. thaliana* natural, several enzymatic decoration genes involved in the modification of core glucosinolate [20,21,22] and flavonoid [23] structures, were characterized by the identification of genomic features indicative of neo-functionalization of tandem duplicated genes. It is important to note that gene duplication is considerably more prominent in plant genomes [24], and that this is a likely cause of the fact that they are also metabolically considerably more diverse than other organisms [25,26]. The fact that such metabolic polymorphism is generally found among natural accessions, but not among related plant species, except occasionally in the case of close relatives such as *Arabidopsis lyrata*, these neo-functionalized genes are generally regarded to be recently evolved genes. As described above, although the core biosynthetic genes of flavonoid production have never been detected as a metabolic gene cluster, genes encoding the decorative reactions were. Such genes are responsible for a major proportion of the diversity of flavonoids. This fact urges caution to pay attention to different aspects of diversity, dependent on the chemical nature of the compounds in question. It furthermore underlines that the investigation of both core and decorative secondary reactions are needed, in order to fully understand the radiance of metabolic evolution. 

In order to understand metabolic evolution, the analysis of neighboring genes in plant genomes would appear to be a sensible starting point. However, since on average, 65% of annotated genes in plant genomes have a duplicate copy [27], the detection of key genomic region and genes of species specific specialized metabolism is highly complex. This has been addressed computationally with the programs PhytoClust [28] and PlantiSmash [29] being highly usable tools that allowed web-based searches for gene clusters. However, unfortunately, their operation appears to have been discontinued. However, all is not lost, since genomic comparison via cross-species comparison can be employed for the detection of specific genes, including gene clusters, as well as neo-functionalized gene pairs. Additionally, the co-expression networks analysis based on the correlation co-efficient between expressed genes in different tissues and growth conditions, is another approach to identify functional gene clusters. Indeed, some neighboring genes in Arabidopsis are co-expressed [30] and, for example, the metabolic cluster genes involved in thalianol and marneral biosynthesis in Arabidopsis are co-regulated [16]. We will return to the mechanism underlying this co-regulation in mechanisms by which clustered genes are co-expressed, below.

Here, we review the current understanding of neighboring metabolic genes, including both metabolic gene clusters and tandem neo-functionalized genes. To discuss the detection of neighboring genes located in the gene cluster and neo-functionalization in model plants, we also evaluated the duplication rate of metabolic genes (cytochrome P450, CYP; 2-oxoglutarate-dependent dioxygenase, 2ODD; terpene synthase, TPS; UDP-sugar dependent glycosyltransferase family 1, UGT1; polyketide synthase, PKS) in the genomes of *A. thaliana*, *Oryza sativa*, *Solanum lycopersicum* and *Lotus japonica*. Furthermore, we screened the transcriptional correlation of neighboring genes in transcriptomics datasets. We also present a case study of the approach of detecting metabolic gene clusters, via co-expression analyses focused on plant specialized metabolism.

## 2. Neighboring Genes of Plant Specialized Metabolism 

### 2.1. Gene Clusters Found in Plant Specialized Metabolism

**Figure 1:**
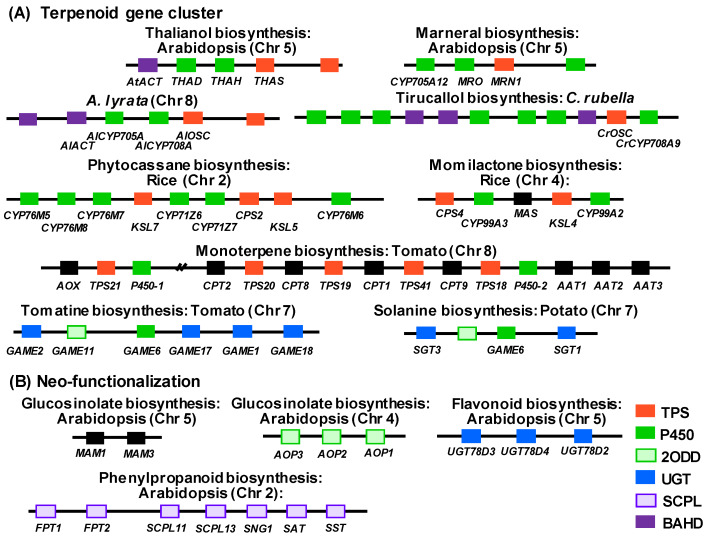
Neighboring genes of plant secondary metabolism. (**A**) Reported terpenoid gene cluster in plants. (**B**) Genomic neo-functionalization in plant secondary metabolism. Abbreviations: ACT, acyltransferase; THAH, thalianol hydroxylase; THAS, thalianol synthase; MRO, putative marneral oxidase; MRN, marneral synthase; baruol synthase, BARS1; OSC, oxidosqualene synthase; CPS, labdadienyl/copalyl synthases; KSL, kaurene synthase-like gene; MAS, momilactone A synthase; SAD, saponin-deficient; AMY, oxidosqualene cyclase; GAME, glycoalkaloid metabolism; SGT, sterol alkaloid glycosyltransferase; AOX, aldehyde oxidase; TPS, terpene synthases; CPT, *cis*-prenyl transferases; AAT, alcohol acyl transferases; TPS, terpene synthase; SCPL, serine carboxypeptidase-like; P450, CYP; 2ODD, 2-oxoglutarate-dependent dioxygenase; UGT, UDP-sugar dependent glycosyltransferase family.

Biosynthetic gene clusters are a genomic region containing at least three different classes of enzymatic genes involved in the same biosynthetic pathway [31]. To date, most of the gene clusters of specialized metabolisms characterized in land plants belong to terpenoid biosynthesis (Figure 1A). In *A. thaliana*, the biosynthesis of two triterpenes, thalianol and marneral were found as metabolic gene clusters [4,15,16]. These clusters contain oxidosqualene cyclase(OSC)-like terpene synthases (thalianol synthase, THAS; marneral synthase, MRN1), P450s (thalian-diol hydroxylase, AtCYP708A2, THAH; thalian-diol desaturase, AtCYP705A5, THAD; AtCYP705A12), BAHD acyltransferase and oxidase (marneral oxidase, MRO) (Figure 1A). The gene cluster located in the genomic synteny of thalianol gene cluster in *A. thaliana* and *A. lyrata* was additionally found in the *Capsella rubella* genome. Interestingly, the structure of this gene cluster in *C. rubella* is diverse and rather, corresponds to the production of tirucallol [17]. This metabolic diversification is created by the functional diversification of both the terpene synthase and P450 in this conserved genomic region. The overexpression of THAS and MRN1 was reported to lead to a dwarf phenotype, whilst the knockout of these genes resulted in longer roots and delayed flowering [4,15]. Importantly, all thalianol biosynthetic genes in both *A. thaliana* and *A. lyrata* showed root-specific gene expression, but tirucallol biosynthetic genes in *C. rubella* displayed bud-specific gene expression pattern. These results suggest that the physiological functions of thalianols and tirucallols are different in plant species, although the origin and evolutional occurrence of their biosynthesis prior to their genetic diversification are predicted to be the same. 

Diterpenoid gene clusters in rice species have been found to be the key genomic region corresponding to the productivity of diverse antifungal-phytoalexins. Biosynthesis of three triterpene-type phytoalexins; phytocassanes, momilactones and oryzalexins were found to be produced by these clusters [9,10,11]. They commonly contain *ent*-CDP (chimera diterpene) synthase type diterpene synthases (CPS), kaurene synthase-like (KSL), and P450s (CYPs) (Figure 1A). Generally, these rice diterpenoid phytoalexins are induced in response to fungal and bacterial infections [10], however, the momilactones are highly accumulated in grain husks. The physiological functions of phytocassanes, momilactones and oryzalexins have been suggested to be different. Importantly, the gibberellin biosynthetic gene cluster, comprising the biosynthetic genes OsCPS1, kaurene synthase (OsKS1), CYP genes (*ent*-kaurene oxidase, OsKO; *ent*-kaurenoic acid oxidase, OsKAO), has a highly similar structure to that of the rice phytoalexin gene cluster. A tomato monoterpene gene cluster containing five terpene synthase genes (SlTPS), *cis*-prenyl transferase (CPTs) and P450s, was similarly identified by the detection of orthologue gene clusters via comparative genomics between *S. lycopersicum*, *S. pennellii*, *S. habrochaites*, *S. pimpinellifolium* and *S. tuberosum* [13] (Figure 1A). Similarly, a steroidal alkaloid gene cluster also accounting for triterpene derived secondary metabolites, that was conserved in both the tomato and potato genomes, was identified [12] (Figure 1A). Furthermore, a second steroidal alkaloid gene cluster was recently observed in a multiomics study of tomato domestication and improvement [32]. All three steroidal alkaloid gene clusters contain P450, 2ODDs and UGTs. 

### 2.2. Neo-functionalization Following Tandem Gene Duplication

The current framework of genetics-based strategies coupled with metabolomic and transcriptomic approaches have been largely performed in functional genomics approaches, aimed at the identification of key genes and genomic regions involved in species- or accession-specific secondary metabolism [7]. Within these studies, considerable neo-functionalization has been observed in the genomic regions originated by tandem duplications in Arabidopsis. In the metabolite profiling of *Arabidopsis thaliana* accessions, the intra-species metabolic polymorphism of glucosinolates was identified with the *AOP2/3* (Aliphatic glucosinolate 2-oxo acid-dependent dioxygenase) and *MAM1/3* (Methylthioalkylmalate synthase 1 and 3) tandem gene duplication region [20,21,22]. Furthermore, the metabolomics analysis of floral secondary metabolites among Arabidopsis natural accessions revealed that flavonol-phenylacyltransferase (AtFPT2) was found as a neo-functionalization of the serine carboxypeptidase-like (SCPL) tandem duplicated genomic region [23]. In this genomic region containing a total of seven SCPL genes, four acyltransferase genes, including AtFPT2, have been experimentally confirmed. These FPT genes have slightly different substrate specificities with regard to their phenylacyl acceptors, but not their phenylacyl donors. Interestingly, a comparative genomics analysis revealed that the tandem gene duplication of FPT genes were different between close Brassica relatives with the exception of *A. lyrata*. These genes are predicted to be the result of neo-functionalization following tandem gene duplication and to have relatively recently evolved, because gene duplication occurred among natural accessions and is not conserved in any but the most closely related Brassica species. Since the decorative reaction catalyzed by AtFPT2 could render much higher tolerance against UV-B irradiance stress, the gene deletion of AtFPT2 is thought to be selected against by natural light stress. This example demonstrates how duplication and neo-functionalization can effectively expand the metabolic diversity of secondary metabolism. However, it only tells part of the story, since protection against light stress in other species has arisen by convergent evolution. Indeed, we recently screened the KNApSAcK database for phenylacylated flavonoids and found that rare Arabidopsis was in no means unique in harboring such compounds [33]. Indeed, one of those phenylacylated flavonols was previously identified to confer UV-B protection to spruce [34]. Similarly, wide screens of the metabolic repertoire of rice and the wild barley quinke revealed that the 5-*O*-glycosylation, 7-*O*-glycosylation and 8-*C*-pentosylation of flavones conferred UV tolerance to these species, respectively [35,36]. However, whilst an interesting aside, these examples indicate the limitations of the approaches we are championing here, rather than highlighting their utility, so we will not dwell on them further. Suffice to say, metabolic profiling of association mapping panels and in particular the checking of decorative modifications [37] may ultimately prove to be a highly effective pre-selection of genomic regions in which to search for either metabolic gene clusters or neo-functionalized genes.

The decorative enzymes of secondary metabolism, such as UGT1, glycoside hydrolase family 1-type gene (BGLU), BEAT (Benzylalcohol-*O*-acetyltransferase)/AHCT (anthocyanin-*O*-hydroxycinnamoyl transferase)/HCBT (anthranilate-*N*-hydroxycinnamoyl/benzoyltransferase)/DAT (deacetylvindoline-4-*O*-acetyltransferase) (BAHD) genes and SCPL genes, are often found next to highly similar “tandem genes”. These decorative enzymatic genes are additionally generally vital in the creation of the chemical diversity inherent in plant secondary metabolism. For example, in Arabidopsis flavonoid biosynthesis, anthiocyanin-3-*O*-glycoside-2″-*O*-phenylacyltransfease [38] and flavonol-3-*O*-glycoside-2″-*O*-phanylacyltransfease [23] were identified in tandem with a BAHD and a SCPL gene, respectively. Such functional convergence of enzymatic properties sometimes renders the identification of such features difficult. For example, the *FTP1* and *FTP2* genes described above were not discussed in the initial definition of the SCPL cluster [39,40] (Figure 1B). Additionally, in some cases of neo-functionalized genes which occur by convergent evolution, the protein sequence does not show a higher similarity between proteins which have exactly the same function (e.g. flavonoid glactocyltransferase of grapevine, kiwi and Vinga) [41,42]. Given the difficulty in such cases, we suggest the adoption of an integrative approach, taking into account protein sequence similarity, as well as proximately alongside transcriptional co-regulation. 

### 2.3. Co-expression Networks of Neighboring Genes for the Discovery of Metabolic Cluster Genes and Neo-functionalized Genes

Co-expression network analysis, which is assessed via the analysis of large-scale transcriptomics data, has greatly aided the elucidation of gene annotation and functional genomics in a broad range of plant species [7,43,44,45]. Co-expression network analysis is well-developed in plant science, however, such multi-gene coefficient based approaches can still be further refined by data optimization strategies, including the use of target-defined sub-datasets [43,46] and targeted gene network analysis [47,48]. Neighboring gene sets found in genome-wide gene annotation have been tested as an approach for the prediction of operon-like gene clusters in the Arabidopsis genome [30,49]. Gene ontology (GO), or KEGG-based target co-expression analysis, revealed that some biosynthetic/catabolic genes in pathways, such as phospholipid degradation and porphyrin and chlorophyll metabolism clusters, are highly correlated gene groups [49]. Such co-expression network analyses are able to identify metabolic gene clusters constructed by genes which are transcriptionally co-regulated in certain tissues and/or stress conditions. In order to carry out a co-expression network approach for neighboring genes, the genomic regions containing putative metabolic gene clusters and tandem gene duplication must first be assigned. For example, a genomic survey of putative specialized metabolic gene clusters in four major model plants (*A. thaliana*, *Oryza sativa*, *Solanum lycopersicum* and *Lotus japonica*) is evaluated. 

The respective genomic regions of putative gene clusters and neo-functionalized gene pairs were defined for five gene families, namely P450, 2ODD, TPS and PKS, and UGT1, which are the cardinal gene families of plant-specialized metabolism (Table 1). In our genomic survey, 109 genomic regions in *A. thaliana*, 163 genomic regions in *O. sativa*, 151 genomic regions in *S. lycopersicum* and 70 genomic regions in *L. japonica*, were found as either gene clusters and/or tandem gene duplications containing more than three genes located in the same genomic region. Amongst these genomic region, single tandem gene duplications of single gene families, indicative of potential neo-functionalized genes, were found in 70 regions in *A. thaliana*; 129 regions in *O. sativa*; 101 regions in *S. lycopersicum* and 52 regions in *L. japonica*. The results of our genomic survey include known metabolic gene clusters, for example, thalianol and marneral biosynthetic genes in Arabidopsis [17,49], steroidal glycoalkaloids in tomato [12], triterpene-type phytoalexin biosynthetic genes [10], cyanogenic glucoside biosynthesis in *L. japonica* [50], as well as novel putative metabolic gene clusters (39 regions in *A. thaliana*; 34 regions in *O. sativa*; 50 regions in *S. lycopersicum* and 18 regions in *L. japonica* (Table 1)).

**Figure 2:**
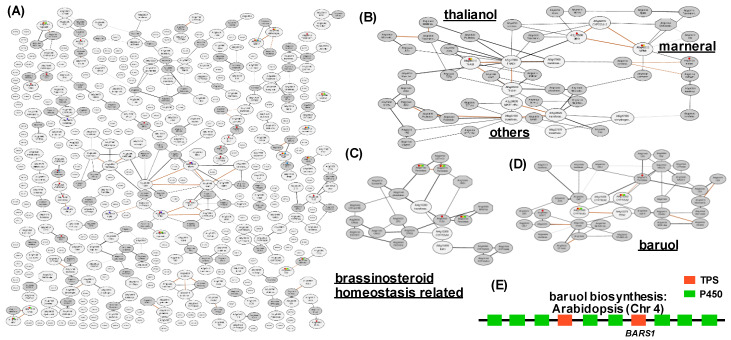
Co-expression analysis of neighboring genes in Arabidopsis genome. (**A**) a global overview of the co-expression of clustered genes in Arabidopsis, (**B**) a co-expression network of terpenoid metabolic gene clusters, (**C**) a co-expression network of baruol synthase, (**D**) a co-expression network of putative brassinosteroids homeostasis related genes, (**E**) putative gene cluster of baruol biosynthesis. Abbreviations: terpene synthases; P450, CYP.

Having the candidates of metabolic gene clusters and neo-functionalized genes in hand, a co-expression approach was conducted. A total of 507 Arabidopsis genes which are putatively annotated as metabolic gene cluster, were used for the co-expression network analysis by ATTED-II [51,52]. Figure 2A provides a global overview of the co-expression of clustered genes in Arabidopsis. Within these co-regulated networks, three networks could be detected as neighboring gene co-expression networks in Arabidopsis. One of the co-expressed neighboring genes that we revealed were terpenoid gene clusters (Figure 2B) of thalianol and marneral biosynthesis, which are known specialized metabolic gene clusters within the Arabidopsis genome. The other co-expressed network contains brassinosteroids inactivator 1 (BIA1) gene involved in brassinosteroid homeostasis (Figure 2C) [53]. Furthermore, a putative gene cluster containing both P450s and TPSs, as well as the gene encoding baruol synthase (BARS1), was detected as a co-expressed metabolic gene cluster-like genomic region (Figure 2D and 2E). Whilst this putative gene cluster will clearly need to be validated experimentally, the fact that we re-found already characterized Arabidopsis gene clusters is highly reassuring. As previously reported [30,49], such a co-expression network approach combined with the genomic survey of neighboring genes is highly useful to identify metabolic gene clusters. It will be interesting in future studies to assess whether it is equally useful at identifying neo-functionalized genes. The ever-increasing wealth of annotated plant genomes, both in quantity and more recently in quality, alongside a similar torrent of transcriptomic datasets, will likely greatly expand the capacity of this approach. Additionally, a cross-species comparative genomics between close relatives or accessions will likely be highly useful to understand the evolution of metabolic neighboring clusters, as recently reported in the cross species comparison of terpenoid biosynthesis in three Brassicaceae species [17]. 

### 2.4. Mechanisms by Which Clustered Genes are Co-Expressed

**Figure 3:**
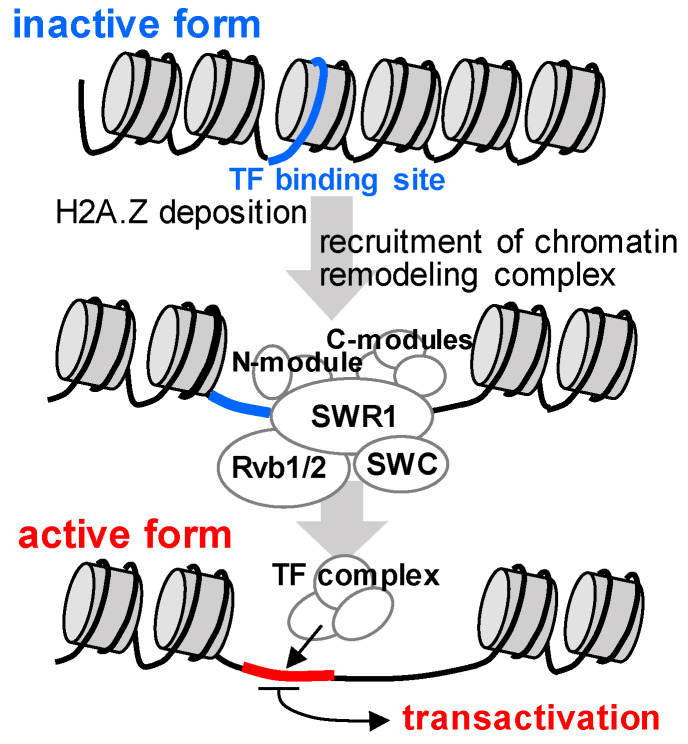
Overview of chromatin remodeling following H2A.Z deposition. N-module and C-module indicate histone and H2A.Z bindings.

In the case of the cross-species comparative genomics approach of triterpene biosynthetic genes in Brassica plant species, the structure of cluster genes conserved in genomic synteny are slightly different between Arabidopsis species and Capsella, for thalianol and tirucallol biosynthesis. Key genes of each pathway were, however, highly co-expressed in a tissue species manner in the respective species [17]. Taking into account the fact that these specialized metabolites are produced in certain tissues and under certain conditions of stress begs the question as to whether there is a mechanistic advantage to their being clustered. Intriguingly, transcription factors regulating plant specialized metabolic gene clusters are still largely unknown. Additionally, the complexity of the regulatory network of combination with miRNA-based post-translational regulation of plant specialized metabolism [54] reported in terpenoid biosynthesis [55] and flavonoid biosynthesis [56], possibly provide the unclear framework of co-expressed network. Indeed despite the fact that some regulators of metabolic gene clusters in bacteria are located in the target gene cluster [57], no such regulators have been reported from surveys of the neighboring genes in known plant clusters. As such, the co-expression network of neighboring genes approach might provide candidate regulators controlling metabolic gene clusters. That said, results from the Osbourn laboratory described that the phylogenetically conserved histone variant, H2A.Z, is essential for the normal expression of the abovementioned thalianol gene cluster of Arabidopsis [16,58]. Indeed, in their study, Nützmann and Osbourn revealed that the levels of thalianol hydroxylase were altered in five of six mutants defective in histone modifications and chromatin remodeling [16,59]. Thus, they indicate that the SWR1 chromatin remodeling complex is required for the incorporation of H2A.Z into the nucleosomes (Figure 3). Indeed, H2A.Z deposition has previously been demonstrated to activate the DAL gene cluster of yeast [60]. Further experiments in both the Arabinol and marenol gene clusters revealed that H2A.Z occupancy into nucleosomes within the gene clusters leads to a localized opening of the chromatin structure, and thereby facilitates cluster expression [16]. This mechanism is illustrated in Figure 3. It is, however, also important to note that chromatin regulation has been reported to affect the synthesis of a number of compounds, including phenylpropanoids, glucosinolates and gibberellins [16,61,62], which are not encoded by cluster genes. Therefore, the use of chromatin marks as a screening strategy for clusters should be approached with caution.

## 3. Concluding Remarks and Future Prospects

Neighboring genes involved in plant specialized metabolism, such as metabolic gene cluster and neo-functionalized genes, are often key in metabolic evolution and metabolic diversification. The distance between biosynthetic genes involved in plant specialized metabolism seems to correlate to the age of the biosynthesis after its occurrence. Therefore, species-specific biosynthesis, namely relatively recent biosynthetic innovations, show much clearer genetic signatures of metabolic evolution. As we report, the subjection of neighboring genes involved in plant specialized metabolism to genomic surveys and co-expression network analysis represents a simple way to find such structural features within plant genomes. Moreover, comparative genomics approaches can be integrated in order to allow translational genomics research. Furthermore, coupling these approaches with recent insights into histone modification and chromatin modelling offers a further route into identifying putative gene clusters. The ever-increasing number of plant genomes alongside massive increases in the amount and availability of transcriptomics data. This fact highly suggests that the approaches we describe here will become increasingly useful in future studies concerned with understanding both the evolution and metabolic regulation of pathways of plant-specialized metabolism.

## Figures and Tables

**Table 1:** Genomic survey for prediction of the metabolic gene clusters and neo-functionalization.

Plant Species	Annotated Metabolic Gene Cluster	Tandem Gene Duplication of Single Gene Family				
		P450	2ODD	TPS	PKS	UGT
*A. thaliana*	39	28	16	6	5	15
*O. sativa*	34	57	15	10	10	37
*S. lycopersicum*	50	30	24	8	9	30
*L. japonicus*	18	29	7	5	4	7
